# Reproductive outcomes in women with unicornuate uterus undergoing in vitro fertilization: a nested case-control retrospective study

**DOI:** 10.1186/s12958-018-0382-6

**Published:** 2018-07-06

**Authors:** Yanrong Chen, Victoria Nisenblat, Puyu Yang, Xinyu Zhang, Caihong Ma

**Affiliations:** 1 Center for Reproductive Medicine, Department of Obstetrics and Gynecology, Peking University Third Hospital, Beijing, 100191 China; 2 National Clinical Research Center for Obstetrics and Gynecology, Beijing, 100191 China; 3 Key Laboratory of Assisted Reproductive, Ministry of Education, Beijing, 100191 China; 4 Beijing, China

**Keywords:** Unicornuate uterus, ART, IVF-ICSI, Reproductive outcomes, Cumulative live birth rate

## Abstract

**Background:**

Unicornuate uterus, a congenital uterine malformation resulting from unilateral maldevelopment of Mullerian duct, is more prevalent in women with infertility. Owing to relative rarity of the condition, the evidence on the associated reproductive outcomes is derived from small heterogeneous studies that report different clinical endpoints and often do not account for the anatomical variations of unicornuate uterus. The aim of this study was to evaluate the embryological and clinical outcomes following IVF-ICSI treatment in women with unicornuate uterus without rudimentary functional cavity (ESHRE-ESGE class IVb).

**Methods:**

Retrospective nested case-control study comprised 342 women with unicornuate uterus and 1026 matched controls who underwent IVF-ICSI cycles between October 2012 and October 2016. Cumulative live birth rate upon one complete IVF cycle, including transfers of all resulting embryos was considered as a primary outcome measure.

**Results:**

Baseline characteristics were comparable between the unicornuate uterus and control groups except for higher rate of primary infertility in unicornuate uterus. Ovarian response to stimulation did not differ between the groups. Transfer of day-3 embryos in fresh cycle resulted in lower clinical pregnancy rate (35.9% vs. 43.9%, *p* = 0.028) and live-birth rate (26.9% vs. 35.2%, *p* = 0.017) per transfer, but the difference was not observed when either cleavage frozen-thaw embryos or blastocysts were transferred. Implantation rate was lower and miscarriage rate was higher in women with unicornuate uterus but the difference between the groups did not reach statistical significance. Transfer of cleavage embryos resulted in significantly higher miscarriage rate and lower live-birth rate in fresh versus frozen-thaw cycles in each group, whereas fresh and frozen-thaw blastocyst embryos had comparable outcomes. Upon completion of one IVF-ICSI cycle, the cumulative pregnancy rate (53.1% vs. 65.7, *p* < 0.001) and cumulative live birth rate (42.4% vs. 54.6%, *p* < 0.001) were significantly lower in women with unicornuate uterus compared to those in women with normal uterus. Cumulative outcomes were superior when embryos were cultured to blastocyst stage.

**Conclusions:**

Women with unicornuate uterus have lower clinical pregnancy and live birth rate after IVF-ICSI treatment compared to women with normal uterus. The treatment outcomes are improved with blastocyst culture, which warrants evaluation in prospective setting.

**Electronic supplementary material:**

The online version of this article (10.1186/s12958-018-0382-6) contains supplementary material, which is available to authorized users.

## Background

Congenital uterine defects represent a wide range of abnormalities of uterine anatomy that result from elongation, fusion and absorption disorders of the bilateral Müllerian ducts during embryogenesis between 6 and 20 weeks of gestation [1]. The prevalence of uterine malformations is estimated at 5.5% in general population and at approximately 8% in women with infertility [2]. Unicornuate uterus is caused by maldevelopment of one Müllerian duct and is relatively uncommon, representing 2.5–13.2% of all uterine malformations [2]. It occurs in 0.1% of unselected population and is more prevalent in women with infertility (0.5%), miscarriage (0.5%) or both (3.1%) [2]. The European Society of Human Reproduction and Embryology–European Society for Gynecological Endoscopy (ESHRE–ESGE) recognizes two types of unicornuate uterus: 1) hemi-uterus with a rudimentary functional contralateral cavity, communicating or non-communicating, due to a partial Mullerian duct development (class IVa) and 2) isolated hemi-uterus caused by a unilateral agenesis of Mullerian duct (class IVb) [3, 4]. Ovarian development usually is not compromised, although ovary on the affected side may be misplaced higher in the abdomen or even absent in rare cases [5]. Coincidental renal anomalies are common and there is an increased risk of developing endometriosis or chronic pain due to hematometra in women with rudimentary horn [5, 6].

It has been widely recognized that unicornuate uterus is associated with increased risk of miscarriage, ectopic pregnancy in rudimentary horn and adverse obstetrics outcomes [5–7]. The relationship between unicornuate uterus and infertility is less clear. A retrospective observational study in 3181 women reported that 23.7% of women with unicornuate uterus were diagnosed with subfertility [8]. A meta-analysis of 25 studies on women with different Mullerian anomalies revealed that a probability of spontaneous conception was not different in women with unicornuate uterus compared with the controls, although these conclusions were based on two small retrospective studies [7]. Several investigators demonstrated poorer outcomes of assisted reproductive technology (ART) treatments in women with unicornuate uterus compared to those with normal anatomy [9–13]. Most of these studies, however, included low number of patients, focused on varying treatment endpoints and presented reproductive outcomes in different ways, all of which challenge the evaluation and synthesis of the reported data. Moreover, none of these studies stratified the outcomes by the type of unicornuate uterus, which may influence reproductive outcomes owing to variations in uterine vascularity, degree of uterine muscle development and presence of other concurrent pelvic pathology [14].

The aim of this study was to evaluate the embryological and clinical outcomes in women with unicornuate uterus without rudimentary horn (ESHRE-ESGE class IVb) following one complete ART cycle including the transfer of all the resulting fresh and frozen-thaw embryos, with a focus on the cumulative birth rate as a primary outcome measure.

## Methods

### Study design and participants

Retrospective nested case control study was conducted at the Reproductive Centre of the Peking University Third Hospital, a tertiary university hospital and a center of excellence in Reproductive Medicine in China. We assessed the medical records of all women who underwent in vitro fertilization (IVF) or intracytoplasmic sperm injection (ICSI) cycles between October 1st, 2012 and October 31st, 2016. Patients were identified through the hospital electronic database.

In addition to the routine pre-treatment work-up, which included history, gynecological examination, baseline reproductive hormones, thyroid function, tubal patency test, pelvic ultrasound and male partner semen analysis, women with any suspected uterine pathology underwent hysteroscopy with or without laparoscopy to confirm the diagnosis and karyotype as deemed appropriate within the clinical context. The inclusion criteria in the study group (unicornuate uterus group) were as follows: 1) unicornuate uterus class IVb (isolated hemi-uterus without functional rudimentary cavity) diagnosed by 3D transvaginal sonography (3D-TVS) and by hysteroscopy, with or without laparoscopy and MRI (Magnetic Resonance Imaging); 2) first IVF/ICSI cycle in our center. The exclusion criteria were: 1) other uterine malformations (septum, unicornuate uterus class IVa, bicornuate uterus); 2) endometrial lesions (polyps, endometrial hyperplasia, intrauterine adhesions); 3) uterine fibroids distorting uterine cavity diagnosed by TVS or hysteroscopy; 4) sonographic features of adenomyosis; 5) chromosomal abnormality of male or female partner; 6) patients who undertook a donor oocyte program or had preimplantation genetic diagnosis (PGD)/preimplantation genetic screening (PGS); 7) patients who had cancelled IVF cycle that did not result in embryo transfer (ET). The control group included women with normal uterus who otherwise met the similar selection criteria and underwent IVF treatment during the study period. Controls were randomly selected from the same database, matched in a ratio of 1:3 by age, BMI, cause of infertility, and number of embryos transferred.

### ART treatment protocols

All participants underwent ovarian stimulation by using either long GnRH agonist downregulation or antagonist protocol, using either recombinant or human menopausal gonadotrophins. Human chorionic gonadotrophin (hCG) trigger was administered when there were at least one to three follicles above 18 mm. Stimulation protocol, type of gonadotrophins and starting dose were at discretion of the treating clinician in discussion with the patient. Ultrasound-guided transvaginal oocyte retrieval was performed 36–38 h after the trigger injection according to the department protocol. Oocytes were inseminated either by conventional IVF or by ICSI depending on sperm quality. Fertilization was assessed 17–19 h after insemination and was defined by the presence of two pronuclears (2PN) and two polar bodies (PBs). An embryo quality was assessed 68–72 h (day 3) after insemination according to the Istanbul Consensus Workshop on Embryo Assessment criteria [15]. Day-3 cleavage embryos were either transferred or cultured for the additional 48 h to the blastocyst stage. The blastocysts were evaluated on day 5 by using Gardner morphological grading system [16]. All spare embryos were cryopreserved for future use. Two cryopreservation methods including vitrification and slow freezing were used.

Day-3 or day-5 embryos were transferred in a fresh cycle with luteal support by vaginal and/or intramuscular progesterone, unless there were medical indications for freezing all the embryos. When the cryopreserved embryos generated from the index stimulation cycle were available, frozen-thaw embryo transfer (FET) was performed in a natural or artificial estradiol and progesterone endometrium priming as described previously [17]. The best morphological grade embryos were selected for transfer and if were not available, the decision to transfer lower quality embryo was based on clinical circumstances and patient wishes after appropriate counselling. ETs were performed by using a soft catheter (K-Soft 5100; Cook, Queensland, Australia). Serum hCG was measured 14 days after ET and was considered positive for hCG level ≥ 10 IU. Transvaginal ultrasonography at 30 days after transfer confirmed clinical pregnancy if intrauterine gestational sac was demonstrated.

### Outcome measures

Cumulative live birth rate was a primary outcome measure and was calculated by the number of first live births generated from a single IVF/ICSI cycle including all the fresh or frozen-thaw ETs generated from the index cycle divided by all women who received treatment. Live birth was defined as a pregnancy that led to delivery of at least one living child, irrespective of the duration of gestation.

Secondary outcomes included the following: 1) implantation rate (the number of gestational sacs divided by the number of transferred embryos); 2) miscarriage rate (loss of clinical pregnancy before 24 weeks of gestation divided by the number of clinical pregnancies); 3) cumulative clinical pregnancy rate (number of first clinical pregnancies from a single IVF/ICSI cycle including all the fresh or frozen-thaw ETs generated from the index cycle divided by all women who received treatment); 4) clinical pregnancy per transfer cycle (number of clinical pregnancies divided by the number of women who had transfer); and 5) live birth rate per transfer cycle (number of live births divided by the number of women who had transfer). The ‘per cycle’ parameters were calculated separately for fresh and frozen-thaw ET cycles.

### Statistical analysis

The Student’s t-test was used for comparison of continuous variables between the groups. The chi-squared test or Fisher’s exact test, where appropriate, were used for comparisons of categorical variables. Results are presented as mean ± standard deviation (SD) or as percentages. The multivariable logistic regression analyses were employed to delineate the independent prognostic risk factors for cumulative live birth rate. Statistical significance was set at a probability (p) value < 0.05 (two-sided). All statistical analyses were performed using SPSS 23.0 software (SPSS, Inc., Chicago, IL, USA).

## Results

Overall 1368 women were included in the study. Of them, 342 patients with unicornuate ESHRE-ESGE class IVb uterus comprised the study group and 1026 women served as controls. There was no significant difference between the groups with respect to age, BMI, baseline FSH, duration and cause of infertility (Table 1). Majority of women in both groups were nulliparous. Primary infertility was significantly more common in women with unicornuate uterus (*p* = 0.005). The characteristics of the ART cycles and the embryology outcomes are summarized in Table 2. There was no difference in number of women assigned to a specific stimulation protocol or in number of ICSI cycles. Both groups did not differ in ovarian response to simulation, had comparable yield of oocytes and of cleavage stage embryos, and had similar fertilization rate. The endometrium on hCG trigger day was significantly thinner in women with unicornuate uterus compared to that in controls, but this difference was of marginal clinical value (10.08 ± 1.57 vs. 10.78 ± 1.55, *p*<0.001).

**Table 1:** Baseline characteristics of the study population

	Unicornuate uterus	Controls	*p*-value
	*n* = 342 women	*n* = 1026 women	
Age (years), mean ± SD	30.53 ± 4.20	30.61 ± 4.28	*p* = 0.784
BMI (kg/m^2^), mean ± SD	22.95 ± 3.36	22.63 ± 2.93	*p* = 0.120
Basal FSH (mIU/ml), mean ± SD	6.21 ± 2.40	5.95 ± 2.59	*p* = 0.089
Primary infertility, n (%)	215/342 (62.9%)	556/1026 (54.2%)	p = 0.005
Infertility duration (years), mean ± SD	4.17 ± 3.24	4.26 ± 3.11	*p* = 0.640
Nulliparity, n (%)	328/342 (95.9%)	961/1026 (93.7%)	*p* = 0.124
Cause of infertility			*p* = 0.891
Tubal factor, n (%)	216/342 (63.2%)	684/1026 (66.7%)	
Male factor, n (%)	63/342 (18.4%)	176/1026 (17.2%)	
Endometriosis, n (%)	5/342 (1.5%)	12/1026 (1.2%)	
PCOS, n (%)	11/342 (3.2%)	26/1026 (2.5%)	
POR, n (%)	13/342 (3.8%)	37/1026 (3.6%)	
Unexplained, n (%)	34/342 (9.9%)	91/1026 (8.9%)	

*BMI* body mass index, *FSH* follicle stimulating hormone, *PCOS* polycystic ovary syndrome, *POR* poor ovarian response

**Table 2:** Characteristics of IVF-ICSI stimulation cycles

	Unicornuate uterus	Control	*p*-value
	*n* = 342 women	*n* = 1026 women	
Number of IVF/ICSI cycles, n	342	1026	
ICSI, n (%)	26.3% (90/342)	30.5% (310/1026)	*p* = 0.146
GnRH agonist protocol, % (n)	55.8% (191/342)	61.0% (626/1026)	*p* = 0.092
Total gonadotrophin dose (IU), mean ± SD	2778.29 ± 866.62	2730.50 ± 1214.06	*p* = 0.501
EM thickness on HCG-day (mm), mean ± SD	10.08 ± 1.57	10.78 ± 1.55	*p*<0.001
E2 on hCG-day (pmol/L), mean ± SD	9661.81 ± 6011.21	9442.79 ± 5834.27	*p* = 0.580
LH on hCG-day (mIU/ml), mean ± SD	2.76 ± 1.42	2.81 ± 1.47	*p* = 0.651
P on hCG-day (nmol/L), mean ± SD	1.73 ± 1.79	1.62 ± 2.91	*p* = 0.526
Oocytes collected, mean ± SD	13.11 ± 7.77	12.28 ± 6.02	p = 0.073
2PN embryos, mean ± SD	7.63 ± 5.88	7.10 ± 4.60	*p* = 0.113
Day-3 embryos, mean ± SD	5.24 ± 5.30	4.95 ± 4.21	*p* = 0.363

*E2* estradiol, *EM* endometrial, *hCG* human chorionic gonadotrophin, *ICSI* intracytoplasmic sperm injection, *IVF* in vitro fertilization, *2PN* two pronuclear zygote, *P* progesterone

Each woman completed one IVF-ICSI treatment including all fresh and frozen-thaw ETs of the embryos generated from this cycle. There was a total of 1939 ET cycles, which included 500 ET cycles in the study group and 1439 ET cycles in controls. The number of embryos transferred per fresh or frozen-thaw cycle, either at cleavage or blastocyst stage was similar between the study and control groups. The clinical outcomes of fresh ETs are presented in Table 3. Cleavage day-3 stage embryos were transferred in majority of fresh ET cycles: 1216 transfers of cleavage stage embryos and 84 transfers of blastocysts. In women with unicornuate uterus transfer of day-3 embryos in fresh cycle was associated with lower rates of clinical pregnancy (35.9% vs. 43.9%, *p* = 0.028), live birth (26.9% vs. 35.2%, *p* = 0.017) and multiple live birth (4.9% vs. 9.7%, *p* = 0.024) compared to those in controls. Implantation rate was also lower (23.7% vs. 28.0%, *p* = 0.073) and miscarriage rate was higher (25.0% vs. 19.7%, *p* = 0.283) in unicornuate uterus but the difference did not reach statistical significance. For fresh cycle blastocyst transfers the comparison between the two groups failed to demonstrate significant difference for any of the outcome parameters. The results of frozen-thaw ET (FET) are illustrated in Table 4. There were 307 cycles of cleavage stage ET and 332 cycles of blastocyst transfer. No significant difference was observed between the study and control groups with respect to any of the clinical endpoints when either cleavage stage or blastocyst embryos were transferred, although there was a similar trend towards lower clinical pregnancy and live birth rate in unicornuate uterus group.

**Table 3:** Reproductive outcomes calculated per fresh ET cycle

	Unicornuate uterus	Control	OR (95% CI)	*p*-value
	*n* = 342 women	*n* = 1026 women		
Cleavage day-3 ET cycles, n	223	993		
Embryos per transfer, mean ± SD	1.87 ± 0.49	1.93 ± 0.46		*p* = 0.123
Implantation rate, % (n)	23.7% (99/418)	28.0% (535/1914)	0.847 (0.703–1.021)	*p* = 0.073
Clinical pregnancy rate, % (n)	35.9% (80/223)	43.9% (436/993)	0.817 (0.676–0.987)	*p* = 0.028
Miscarriage rate, % (n)	25.0% (20/80)	19.7% (86/436)	1.267 (0.829–1.937)	*p* = 0.283
Live birth rate, % (n)	26.9% (60/223)	35.2% (350/993)	0.763 (0.605–0.963)	*p* = 0.017
Multiple live birth rate, % (n)	4.9% (11/223)	9.7% (96/993)	0.510 (0.278–0.936)	*p* = 0.024
Blastocyst ET cycles, n	59	25		
Embryos per transfer, mean ± SD	1.07 ± 0.25	1.16 ± 0.37		*p* = 0.192
Implantation rate, % (n)	34.9% (22/63)	34.5% (10/29)	1.013 (0.553–1.853)	*p* = 0.967
Clinical pregnancy, % (n)	37.3% (22/59)	40.0% (10/25)	0.932 (0.520–1.670)	*p* = 0.815
Miscarriage rate, % (n)	18.2% (4/22)	20.0% (2/10)	0.909 (0.198–4.173)	*p* = 0.903
Live birth rate, % (n)	30.5% (18/59)	32.0% (8/25)	0.953 (0.479–1.899)	*p* = 0.892
Multiple live birth rate, % (n)	3.4% (2/59)	0	0.966 (0.921–1.013)	*p* = 0.351

*ET* embryo transfer, *CI* confidence interval, *OR* odds ratio

**Table 4:** Reproductive outcomes per FET cycle

	Unicornuate uterus	Control	OR (95% CI)	*p*-value
	*n* = 342 women	*n* = 1026 women		
Cleavage day-3 FET cycles, n	93	214		
Embryos survival rate, % (n)	90.2% (203/225)	91.9% (475/517)		*p* = 0.478
Embryos per transfer, mean ± SD	2.18 ± 0.59	2.21 ± 0.68		*p* = 0.743
Implantation rate, % (n)	16.7% (34/203)	20.4% (97/475)	0.820 (0.575–1.169)	*p* = 0.267
Clinical pregnancy, % (n)	32.3% (30/93)	35.5% (76/214)	0.908 (0.643–1.283)	*p* = 0.581
Miscarriage rate, % (n)	6.7% (2/30)	9.2% (7/76)	0.724 (0.159–3.288)	*p* = 0.672
Live birth rate, % (n)	30.1% (28/93)	32.2% (69/214)	0.934 (0.648–1.346)	*p* = 0.712
Multiple live birth rate, % (n)	4.3% (4/93)	9.8% (21/214)	0.438 (0.155–1.241)	*p* = 0.105
Blastocyst FET cycles, n	125	207		
Embryos survival rate, % (n)	97.9% (141/144)	98.7% (234/237)		*p* = 0.677
Embryos per transfer, mean ± SD	1.13 ± 0.34	1.13 ± 0.34		*p* = 0.949
Implantation rate, % (n)	34.0% (48/141)	36.8% (86/234)	0.926 (0.697–1.231)	*p* = 0.596
Clinical pregnancy, % (n)	36.8% (46/125)	40.1% (83/207)	0.918 (0.619–1.219)	*p* = 0.550
Miscarriage rate, % (n)	21.7% (10/46)	10.8% (9/83)	2.005 (0.878–4.576)	*p* = 0.094
Live birth rate, % (n)	28.8% (36/125)	35.7% (74/207)	0.806 (0.579–1.121)	p = 0.192
Multiple live birth rate, % (n)	1.6% (2/125)	1.4% (3/207)	1.104 (0.187–6.516)	*p* = 0.913

*FET* frozen embryo transfer, *CI* confidence interval, *OR* odds ratio, *SD* standard deviation

The results of the comparisons between fresh and frozen-thaw ETs stratified by the type of embryos transferred in each group is summarized in Additional file 1: Table S1. Transfer of day-3 embryos in fresh cycle resulted in significantly higher implantation and clinical pregnancy rate, but significantly lower miscarriage rate than in frozen-thaw cycle in both the unicornuate uterus or control groups. The outcomes of the blastocyst transfers were not different between the fresh and frozen-thaw cycles in either group.

The cumulative outcomes of one complete ART treatment with all the ETs generated from a single stimulation-oocyte retrieval cycle are presented in Table 5. The cumulative clinical pregnancy rate was significantly lower in women with unicornuate uterus than that in controls (53.1% vs. 65.7%, *p* < 0.001). Likewise, the unicornuate uterus group had significantly lower cumulative live birth rate compared to the women with normal uterine anatomy (42.4% vs. 54.6%, *p* < 0.001) (Table 5A). Among women with unicornuate uterus, transfer of cleavage embryos resulted in significantly lower clinical pregnancy and live birth than transfer of blastocyst embryos (47.5% vs. 65.1%, *p* = 0.006 and 38.2% vs. 53.0%, *p* = 0.021), respectively (Table 5B).

**Table 5:** Cumulative reproductive outcomes from one complete ART cycle including fresh and frozen-thaw ETs

A. Cumulative outcomes in women with unicornuate uterus and in controls^a^				
	Unicornuate	Control	OR (95% CI)	*p*-value
	*n* = 335 women	*n* = 920 women		
Number of IVF/ICSI cycles, n	335	920		
Number of ET cycles, n	486	1206		
Fresh ET cycles, n	277	916		
FET cycles, n	209	290		
Cumulative pregnancy rate, % (n)	53.1% (178/335)	65.7% (604/920)	0.809 (0.724–0.904)	*p*<0.001
Cumulative live birth rate, % (n)	42.4% (142/335)	54.6% (502/920)	0.777 (0.677–0.892)	*p*<0.001
B. Cumulative outcomes in women with unicornuate uterus group stratified by the type of embryos transferred^b^				
	Cleavage day-3 ET	Blastocyst ET	OR (95% CI)	*p*-value
	*n* = 217 women	*n* = 83 women		
Number of IVF-ICSI cycles, n	217	83		
Number of ET cycles, n	251	148		
Fresh ET cycles, n	185	64		
FET cycles, n	66	84		
Cumulative pregnancy rate, % (n)	47.5% (103/217)	65.1% (54/83)	0.730 (0.591–0.901)	*p* = 0.006
Cumulative live birth rate, % (n)	38.2% (83/217)	53.0% (44/83)	0.722 (0.554–0.939)	*p* = 0.021

*ART* assisted reproductive technologies, *CI* confidence interval, *ET* embryo transfer, *FET* frozen-thaw ET, *ICSI* intracytoplasmic sperm injection, *IVF* in vitro fertilization, *OR* odds ratio

^a^only women who either achieved pregnancy or utilized all the embryos resulting from the index stimulation cycle were included in this analysis

^b^ only women who had one type of embryos transferred in both fresh and FET cycles, either only cleavage or only blastocysts, were included in this analysis

Our study used the cumulative live birth as a dependent variable, unicornuate uterus, infertility type, protocol type, basal FSH and oocytes collected as independent variables, analyzed by the method of multivariable logistic regression. The unicirnuate uterus (OR 0.756, 95%CI 0.586–0.974, *p* = 0.030) and infertility type (OR 0.487, 95%CI 0.391–0.607, *p* < 0.001) were the independent factors of the cumulative live birth.

## Discussion

This study aimed to evaluate the IVF-ICSI treatment outcomes in infertile women with unicornuate uterus without functional rudimentary cavity. We considered cumulative live birth rate after one cycle of ovarian stimulation - oocyte retrieval with all the resulting fresh and frozen-thaw ET cycles as a primary outcome measure and reported the data from 342 women with unicornuate uterus and 1026 randomly selected matched controls from the same cohort. To the best of our knowledge, this is the largest report that includes well-characterized women with specific phenotype of unicornuate uterus and accounts for multiple confounding factors that may impact the treatment outcomes.

Our results demonstrate that unicornuate uterus did not affect ovarian response to stimulation and embryology outcomes. Overall the IVF-ICSI endpoints in women unicornuate uterus without functional rudimentary cavity are reassuring but are inferior to those in women with normal uterus. In women with unicornuate uterus, the odds of achieving clinical pregnancy following one complete IVF-ICSI cycle were 24% lower (OR 0.809, 95%CI 0.724–0.904) and the odds of live birth were 28% lower (OR 0.777, 95% CI 0.677–0.892) than in women with normal uterine anatomy after control for important demographic and clinical confounders.

This is in line with the results of previous observational studies that demonstrated lower pregnancy rate and/or live-birth in women with unicornuate uterus than in controls with normal uterine morphology [6, 10, 11, 18]. In contrast, Jayaprakasan et al., did not observe difference in ART outcomes between women with uterine malformations and normal uterus, but the study included only 6 women with bicornuate uterus, while arcuate uterus represented majority of the evaluated uterine malformations [13]. When we stratified the outcomes by the type of embryos transferred, statistically significant difference between the groups with respect to clinical pregnancy and live birth were observed only for fresh cleavage stage embryos, while transfer of frozen-thaw cleavage or of blastocyst embryos resulted in only non-significantly lower outcomes in unicornuate uterus group.

In contrast with previously demonstrated lower implantation rate in unicornuate uterus [18], in this study implantation rate was only non-significantly reduced in women with unicornuate uterus, which is in agreement with the findings reported by Ozgur et al. [10]. The association between unicornuate uterus and miscarriage following either natural or assisted conception were previously reported by several investigators [6, 10, 11, 13, 18, 19]. In this study, there was only non-significant increase in miscarriage rate in the overall group of women with unicornuate uterus. However, women who had a transfer of fresh cleavage stage embryos had significantly higher miscarriage rate and lower live-birth rate compared to those who had frozen-thaw cycles, which held true for women with either unicornuate or normal uterus. This observation is consistent with the results of recent randomized controlled trial (RCT) that demonstrated that frozen ET cycles were associated with higher rate of live birth and lower rate of miscarriage in women with PCOS [20]. Transfer of blastocyst stage embryos did not appear to be associated with different outcomes in either group. Importantly, however, the cumulative reproductive outcomes in women with unicornuate uterus were significantly higher when embryos were cultured to the blastocyst stage with 37% higher odds to achieve clinical pregnancy (OR 0.730, 95%CI 0.591–0.901) and 39% higher odds to achieve live-birth (OR 0.722, 95%CI 0.554–0.939) compared to the transfer of cleavage stage embryos. Improved implantation and pregnancy rates have been increasingly reported for transferred blastocysts compared to cleavage embryos in IVF cycles. This finding should be interpreted with caution in view of relative paucity of blastocyst ET cycles in this study. The most recent Cochrane library systematic review concluded that in general IVF population, the live birth rate per fresh transfer was significantly higher with blastocyst culture compared to cleavage stage embryos, whereas there was no difference between the groups in cumulative live birth rates following both fresh and frozen-thaw cycles resulting from one egg collection [21]. Higher likelihood of failure to make embryo transfer along with lower availability for surplus embryos with blastocyst culture are possible explanation. This meta-analysis, however, included earlier studies employing slow freezing and overall reported low quality evidence for the cumulative outcomes, hence the effect of the day of transfer on cumulative pregnancy and live birth rate remains unclear. In this study women with unicornuate uterus had higher rate of primary infertility, similarly to the observed in previous studies [6]. Unicornuate uterus has been recognized in association with adverse obstetrics and neonatal outcomes, including preterm labor, fetal malpresentation, fetal growth restriction and perinatal death [5–7]. The possible causative factors include reduced uterine muscle mass, aberrant uterine vasculature and smaller size of uterine cavity [5, 14]. The underlying mechanism by which unicornuate uterus affects fertility and ART treatment outcomes is unclear. It has been proposed that derangements in endometrial vascularization with deleterious effect on endometrial receptivity and implantation could play a role [14]. It is also possible that mechanical factor is more likely to be present in unicornuate uterus due to presence of a single tube. Unicornuate uterus has been also associated with increased risk of ectopic pregnancy and endometriosis, particularly in women with functional rudimentary uterine cavity. In our cohort, tubal factor accounted for majority of causes of infertility, 63.2%, which is higher than the estimated ~ 18% in general population of women who undergo ART [22].

The strengths of this study are in its relatively large sample size of well-characterized cohort and the utilized strategies to control for confounding, including carefully 1:3 matched controls and multiple subgroup analyses. In addition, the study reports the cumulative success rate after one complete stimulation cycle, which is a more appropriate way to estimate the ART treatment outcomes than presenting a data per an individual transfer cycle. The information presented this study helps to refine patient counselling and directs the clinicians towards more effective tailored interventions.

The main limitations of this study are its retrospective nature and no information on the obstetrics and neonatal outcomes. Furthermore, not all women that commenced ART treatment in 2016 utilized their available embryos from the index stimulation cycle and were not included in estimation of cumulative live birth rate. Finally, culture to blastocyst was performed in relatively small number of treatment cycles as the shift towards blastocyst embryo transfer has occurred in our center in the last several years.

## Conclusion

In summary, women with unicornuate uterus have lower clinical pregnancy and live-birth rate after IVF-ICSI treatment compared to women with normal uterine morphology with similar baseline characteristics. Culture to blastocyst is associated with the improved treatment cycle outcomes. The findings of this study require further validation in large well-defined cohort of women with unicornuate uterus in different population. It is unrealistic to expect a single-center interventional RCT that focuses on management of unicornuate uterus as the condition is infrequent. Multi-center initiatives or non-randomized prospective studies would help to evaluate the contribution of blastocyst transfer, especially single blastocyst transfer approach to improvement of ART treatment outcomes in women with unicornuate uterus.

## Additional file

Additional file 1: **Table S1.** Reproductive outcomes - comparison between fresh and frozen-thaw ET cycles in each subgroup. (DOCX 20 kb)
