# Supplementary material for: Reproductive outcomes in women with unicornuate uterus undergoing in vitro fertilization: a nested case-control retrospective study
Source: Reprod Biol Endocrinol. 2018 Jul 6;16:64. doi: 10.1186/s12958-018-0382-6 (PMC6034283; doi:10.1186/s12958-018-0382-6)
Supplement: Supplementary file 1 — Table S1. Reproductive outcomes - comparison between fresh and frozen-thaw ET cycles in each subgroup. (DOCX 20 kb) [file 12958_2018_382_MOESM1_ESM.docx]

**Additional file 1: Table S1** Reproductive outcomes - comparison between fresh and frozen-thaw ET cycles in each subgroup

|  | Fresh ET | FET | p-value |
| --- | --- | --- | --- |
| **Unicornuate uterus day-3 ET cycles, n** | **223** | **93** |  |
| Embryos per transfer, mean ± SD | 1.87 ± 0.49 | 2.18 ± 0.59 | p<0.001 |
| Implantation rate, % (n) | 23.7% (99/418) | 16.7% (34/203) | p=0.048 |
| Clinical pregnancy, % (n) | 35.9% (80/223) | 32.3% (30/93) | p=0.539 |
| Miscarriage rate, % (n) | 25.0% (20/80) | 6.7% (2/30) | p=0.032 |
| Live birth rate, % (n) | 26.9% (60/223) | 30.1% (28/93) | p=0.563 |
| **Unicornuate uterus blastocyst ET cycles, n** | **59** | **125** |  |
| Embryos per transfer, mean ± SD | 1.07 ± 0.25 | 1.13 ± 0.34 | p=0.223 |
| Implantation rate, % (n) | 34.9% (22/63) | 34.0% (48/141) | p=0.903 |
| Clinical pregnancy, % (n) | 37.3% (22/59) | 36.8% (46/125) | p=0.949 |
| Miscarriage rate, % (n) | 18.2% (4/22) | 21.7% (10/46) | p=0.734 |
| Live birth rate, % (n) | 30.5% (18/59) | 28.8% (36/125) | p=0.812 |
| **Controls day-3 ET cycles, n** | **993** | **214** |  |
| Embryos per transfer, mean ± SD | 1.93 ± 0.46 | 2.21 ± 0.68 | p<0.001 |
| Implantation rate, % (n) | 28.0% (535/1914) | 20.4% (97/475) | p=0.001 |
| Clinical pregnancy, % (n) | 43.9% (436/993) | 35.5% (76/214) | p=0.024 |
| Miscarriage rate, % (n) | 19.7% (86/436) | 9.2% (7/76) | p=0.028 |
| Live birth rate, % (n) | 35.2% (350/993) | 32.2% (69/214) | p=0.402 |
| **Controls blastocyst ET cycles, n** | **25** | **207** |  |
| Embryos per transfer, mean ± SD | 1.16 ± 0.37 | 1.13 ± 0.34 | p=0.683 |
| Implantation rate, % (n) | 34.5% (10/29) | 36.8% (86/234) | p=0.811 |
| Clinical pregnancy, % (n) | 40.0% (10/25) | 40.1% (83/207) | p=0.993 |
| Miscarriage rate, % (n) | 20.0% (2/10) | 10.8% (9/83) | p=0.397 |
| Live birth rate, % (n) | 32.0% (8/25) | 35.7% (74/207) | p=0.711 |

ET – embryo transfer, FET - frozen embryo transfer, SD – standard deviation
